# Estimation of Kenaf seedling canopy coverage in saline soil using semantic segmentation of UAV RGB images

**DOI:** 10.3389/fpls.2026.1747657

**Published:** 2026-02-10

**Authors:** Wei Wang, Kunzhi Cao, Guiying Luo, Ruohan Huang, Jihao Nie, Junyu Zhang, Jianning Lu, Guoxian Cui, Xia An, Wei She

**Affiliations:** 1 College of Agriculture & Ramie Research Institute, Hunan Agricultural University, Yuelushan Laboratory, Changsha, China; 2 Zhejiang Xiaoshan Institute of Cotton & Bast Fiber Crops, Zhejiang Institute of Landscape Plants and Flowers, Zhejiang Academy of Agricultural Sciences, Hangzhou, China

**Keywords:** deep learning, Kenaf, saline soil tolerance, UAV remote sensing, U-net

## Abstract

The growth status of Kenaf (*Hibiscus cannabinus* L.) seedlings directly impacts its yield and quality. Addressing the challenges of inefficient monitoring and quantitative assessment of Kenaf seedlings under saline-alkali conditions, this study developed an automated method for plant identification and canopy coverage estimation during the seedling stage. This approach leverages high-resolution visible light imagery captured by unmanned aerial vehicles (UAVs) combined with deep learning semantic segmentation techniques. First, a UAV imagery dataset of Kenaf seedlings was constructed through geometric and radiometric calibration, image cropping, and sample annotation. Subsequently, three classical semantic segmentation models—FCN, U-Net, and DeepLabV3+—were trained and compared using image enhancement strategies. Model performance was quantitatively evaluated using metrics including Intersection over Union (IoU), accuracy, precision, and F1 score. Results indicate that all three models effectively segmented Kenaf plants from soil backgrounds. U-Net demonstrated optimal overall accuracy and detail retention, DeepLabV3+ exhibited advantages in small-scale object recognition, while FCN offered high computational efficiency, making it suitable for applications demanding real-time processing. Building upon this, the U-Net architecture was enhanced by incorporating a Self-Attention (SE) channel mechanism, further improving model performance to achieve an IoU of 85.99% and an Dice of 92.44%. Based on segmentation results from the enhanced UNet, plant canopy coverage during the Kenaf seedling stage was calculated. Combined with measured dry bark yield per mu, this enabled analysis of growth performance across varieties under saline-alkali conditions, identifying Xiao 3, K5, and Xiao 2 as materials exhibiting strong saline-alkali adaptability. The study demonstrates that this method enables high-precision identification and quantitative analysis of Kenaf seedlings, providing effective technical support for monitoring seedling growth and variety selection in saline-alkali soils.

## 1 Introduction

Kenaf (Hibiscus *cannabinus L*.), belonging to the genus Hibiscus within the Malvaceae family, is a significant annual bast fiber crop widely utilized in hemp textiles, papermaking, and construction materials (Webber et al., 2002). In recent years, soil salinization has become increasingly severe, emerging as one of the primary factors constraining sustainable agricultural development (Rahman et al., 2002). Therefore, the rational utilization of saline-alkali soils and the selection of salt-tolerant crop varieties are crucial for ensuring agricultural production (Hassani et al., 2021). The adaptability of Kenaf to saline-alkali conditions during the seedling stage directly impacts its growth, development, and ultimate yield, with the emergence rate being particularly sensitive to soil salinity levels (Chen et al., 2024). In modern agricultural production, the rapid and accurate acquisition of crop seedling condition information is a critical step in achieving modernized and precision management of farmland (Lamichhane et al., 2018). The survey methods of traditional crop condition primarily rely on manual measurements and visual interpretation, which are time-consuming and labor-intensive, making them ill-suited for efficient monitoring of large-scale farmland. With the rapid advancement of unmanned aerial vehicle (UAV) remote sensing technology, the acquisition of high-resolution visible light imagery using lightweight, low-cost drones has become a vital method for crop seedling condition monitoring (Zeng et al., 2025). This approach provides new technical support for monitoring the early growth stages of economic crops like Kenaf and studying their adaptability to saline-alkali conditions.

Accurate extraction of crop information from imagery, enabling automated recognition and segmentation, is a critical step in achieving precise monitoring. Image segmentation primarily relies on features such as color, texture, and shape to segment and identify regions of interest (Sadashivan et al., 2021). Color threshold-based segmentation methods utilize differences in the distribution of subjects and backgrounds across grayscale, RGB, and HSV histograms (Feng et al., 2020). achieved high accuracy of 97.4% by segmenting plants and backgrounds using HSV and CieLab (luminance, green-red channel, blue-yellow channel) color spaces (Riehle et al., 2020). Another approach employs unsupervised clustering based on pixel color or texture features (Jia et al., 2020), dividing images into distinct categories from which vegetation regions are extracted. These methods can achieve certain results when the soil background is relatively simple. However, in the face of complex and variable field environments and factors such as weed interference, the color difference between plants and their background is often not clearly discernible (Guo et al., 2025). Relying solely on manual interpretation or traditional image processing methods often leads to misclassification or missed segmentation, making it difficult to achieve precise crop identification.

In recent years, with the rapid advancement of deep learning technology, image segmentation methods based on convolutional neural networks (CNNs) have been widely applied in the field of remote sensing (Kattenborn et al., 2021). Compared to traditional approaches, deep learning models can automatically learn multi-level features from massive datasets, significantly enhancing the accuracy and robustness of crop identification and segmentation. Common semantic segmentation networks such as FCN (Fully Convolutional Network), UNet, SegNet, and the DeepLab (Ronneberger et al., 2015; Wang et al., 2022a; Wang et al., 2022b) series have demonstrated outstanding performance in tasks including crop identification (Lapkovskis et al., 2025), pest and disease detection (Zhang et al., 2022), and canopy cover estimation (Yao et al., 2025). Alkhudaydi employed FCN and transfer learning for semantic segmentation of wheat ear images, providing an effective method for yield trait assessment with an average accuracy exceeding 76% (Alkhudaydi et al., 2019). Jiang combined K-means clustering with an augmented UNet network to perform dual segmentation of rapeseed and weeds, significantly improving recognition accuracy in complex field environments (Jiang et al., 2025). Furthermore, Ding achieved precise segmentation of apple leaf lesions by refining the DeepLabv3+ model, attaining a segmentation accuracy of 98.45% and significantly improving disease detection accuracy (Ding et al., 2025).

Beyond CNN-based models, Transformer-based models and hybrid CNN–Transformer architectures have garnered increasing attention in recent years due to their robust capabilities in modelling long-range dependencies and capturing multi-scale contextual features. For instance, segmentation frameworks based on Vision Transformers (ViT) (Dosovitskiy et al., 2020) and their improved variants, such as EPEMMSA-ViT (Wang et al., 2024) and MMVT (Lucas et al., 2025), have demonstrated outstanding performance in agricultural remote sensing applications for pest and disease identification and soil health assessment. Concurrently, hybrid models integrating CNN’s local perception with Transformer’s global modelling advantages continue to emerge. Examples include FTransUNet (Ma et al., 2024), the lightweight TinySegformer (Zhang and Lv, 2024), and CTFFNet (Jin et al., 2024), which have made significant strides in enhancing sensitivity to small targets and robustness in complex backgrounds. Nevertheless, despite their outstanding performance across multiple agricultural vision tasks, these methods remain under-systematically applied and validated in UAV image segmentation during the crop seedling stage. The small scale and indistinct boundaries of seedlings during this stage impose heightened demands on model stability and generalisation capabilities, areas where existing research remains insufficient. In summary, the evolution from CNNs to Transformers and their hybrid architectures provides powerful tools for accurately extracting crop spatial distribution and early growth information from UAV imagery.

This study aims to utilize drone-captured visible light imagery and semantic segmentation technology to achieve automated identification and precise segmentation of Kenaf seedlings in saline-alkali soils, thereby constructing an efficient and stable model for extracting seedling condition information. By analyzing imagery of young Kenaf seedlings from different varieties within saline-alkali plots, the research explores the response characteristics of Kenaf seedlings to saline-alkali stress during their early growth stage and assesses the salt tolerance adaptability of various cultivars. This provides technical support for selecting salt-tolerant Kenaf varieties and promoting the rational utilization of saline-alkali lands.

To achieve the aforementioned objectives, this paper primarily undertakes the following research activities:

(1)UAV Image Acquisition and Data Preprocessing: Acquire high-resolution visible light imagery of Kenaf seedlings across different saline-alkali plots via UAV flights. Generate complete orthorectified images through geometric correction, radiometric calibration, and subsequent steps. Construct a Kenaf seedling imagery dataset suitable for deep learning segmentation tasks via image cropping and feature annotation.
(2)Construction of an Image Semantic Segmentation Model for Kenaf Seedling Stages: Based on three deep learning segmentation networks—FCN, UNet, and DeepLabv3+—we designed and trained a model suitable for image segmentation of Kenaf seedling stages.
(3)Model Optimization and Evaluation: Enhance model performance through data augmentation techniques, and conduct quantitative analysis and model validation using precision evaluation metrics such as IoU, F1-score, and Precision.
(4)Extraction and Analysis of Kenaf Seedling Information in Saline-Alkali Soils: Utilizing the most effective segmentation model to extract seedling emergence data during the Kenaf seedling stage, investigating the adaptability of different Kenaf varieties to saline-alkali soils, analyzing the relationship between adaptability and final yield, and identifying Kenaf varieties with breeding potential.

## 2 Materials

### 2.1 Experimental area

**Figure 1:**
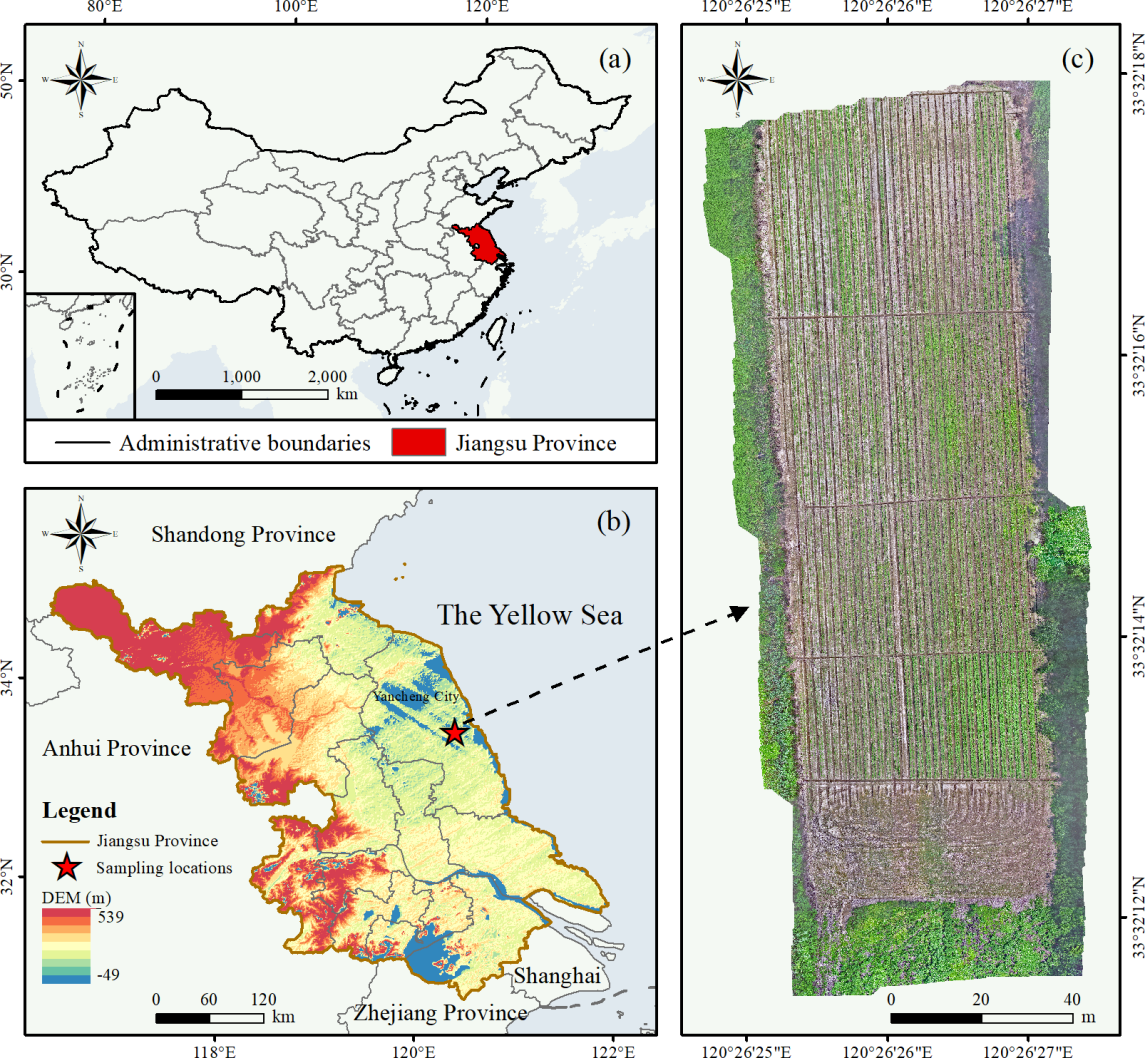
Experimental area map. The red box in **Figure 1c** indicates the effective range ultimately selected from the sample plot.

The experiment was conducted in Yancheng City, Jiangsu Province, China (32°34′N–34°28′N, 119°27′E–120°54′E). Located in the core region of the Yangtze River Delta, Yancheng features flat terrain and a temperate climate, transitioning from the northern subtropical to warm temperate zones. Bordering the East China Sea, the region encompasses approximately 7 million mu (473, 333 hectares) of tidal flats with widespread saline-alkali soils. The experimental area exhibits elevated soil salinity levels, imposing stress on crop germination and early growth (**Figure 1**).

Soil pH ranged from 8.88 to 9.07, with total water-soluble salts (TSW) content between 0.12% and 2.86%. Kenaf was sown using broadcast seeding, with seeds evenly distributed across each experimental plot. Each plot measured 40 m in length and 1.4 m in width. Eleven test varieties were included: H368, Xiao 1, Xiao 2, Xiao 3, K1, K2, K3, K4, K5, K6, and K7.

### 2.2 UAV image acquisition

Visible light imagery was captured using a quadcopter UAV (DJI Inspire 2, DJI Innovation, Guangdong, China) equipped with an RGB camera featuring a resolution of 5472 × 3648 pixels. Multispectral imagery acquisition employed a quadcopter (DJI Phantom 4 Multispectral, DJI Innovation, Guangdong, China) equipped with one visible light sensor and five multispectral sensors. Flight operations occurred from 10:00–11:00 on July 9, 2023, with aerial photography parameters set as follows: flight altitude 25 m, lens tilt angle −90°, flight speed 6 m/s, and both fore-and-aft and sideways overlap rates set to 75%. To minimize image distortion and target movement, data collection was conducted under clear, windless weather conditions. Ultimately, 335 high-resolution visible images and 1, 734 multispectral images were acquired during the Kenaf seedling stage. Image calibration and stitching were completed using DJI Terra software.

### 2.3 Ground-based Kenaf growth data

Prior to harvesting, ground growth data were collected at the Kenaf maturity stage. Fresh plant weight was determined by randomly selecting 20 Kenaf plants and summing their fresh weights; plant height and stem diameter were each taken as the average value from the same batch of 20 plants; bark thickness measurements were obtained by stacking the bark layers from five selected plants to ensure more stable and representative data.

### 2.4 Hardware and software environment

All image processing and deep learning experiments were conducted on the same high-performance workstation. The hardware configuration includes a 13th-generation Intel Core i5-13490F processor (base frequency 2.5 GHz), 64 GB DDR4 memory, and an NVIDIA GeForce RTX 4070 graphics card. The software environment utilized Anaconda-Miniconda3 to build isolated virtual environments running Python 3.8, CUDA 11.3, and PyTorch 1.10. Development and debugging were performed using PyCharm Community Edition 2024. Remote sensing feature extraction was conducted with phenoAI air (AgriBrain, Nanjing, China). This hardware and software configuration provided stable and efficient computational support for model training and testing.

## 3 Methods

### 3.1 Construction of image dataset

Orthoimages were cropped based on neighborhood boundaries to uniform widths and lengths, yielding sub-images of 340 × 512 pixels (Image A) and 340 × 8880 pixels (Image B: single-image representation of a single Kenaf variety). From image A, 600 images were randomly selected and manually annotated using LabelMe software to generate binary semantic segmentation labels for Kenaf seedlings/background. To enhance model robustness and generalization, multiple data augmentation strategies were employed during training, including:

Scale change: Images were scaled at six levels (0.5×, 0.75×, 1.25×, 1.5×, 1.75×, 2×) and uniformly cropped to 512×512 pixels;

Geometric flip: Including horizontal flipping and vertical flipping by 180°;

Photometric distortion: Random adjustments to brightness, contrast, and saturation.

To ensure image quality and consistency, all images underwent mean and standard deviation normalization during dataset construction to minimize color bias caused by lighting variations. Background pixels were filled with 0, while segmented label regions were filled with 255 to ensure label consistency. Finally, the dataset was divided into training, validation, and test sets at an 8:1:1 ratio, providing ample data support for deep learning model construction and evaluation.

### 3.2 Semantic segmentation model construction and training methods

The objective of this study is to establish a standardised, reproducible workflow for monitoring and segmenting Kenaf seedlings using UAV. A systematic comparison will be made of the performance of representative classical semantic segmentation models on this task. In selecting architectures for this study, three were selected based on the following principles.

#### 3.2.1 Model selection rationale

FCN represents a pioneering model that initially accomplished end-to-end pixel-level prediction, thereby establishing the theoretical foundation for all subsequent networks (Long et al., 2015).

U-Net: This architecture is distinguished by its ability to achieve fine-grained structure recovery through skip connections, thus establishing itself as one of the most widely applied and consistently stable architectures in agricultural image segmentation. In particular, it has been demonstrated to be highly effective for small object detection via UAVs (Chen et al., 2018).

DeepLabv3+: The model utilises dilated convolutions and the Atrous Spatial Pyramid Pooling (ASPP) module to deliver robust multi-scale contextual modelling capabilities, thus representing a high-precision segmentation model in complex field environments (Ronneberger et al., 2015).

The second point to consider is the alignment with UAV agricultural imagery characteristics and research task requirements.

Kenaf seedlings in UAV imagery manifest characteristics of small volume, dispersed distribution, fine and irregular edges, while being significantly affected by variations in illumination, shadow interference, and complex background textures. In the domain of agricultural remote sensing, U-Net and DeepLab architectures have been extensively validated for the segmentation of small objects, while FCN serves as a natural baseline model for systematic comparison. Moreover, given that transformer-based models generally necessitate more substantial training datasets and greater computational resources, the present study concentrates on the aforementioned classical and efficient convolutional network architectures to guarantee reproducibility and stability.

In the domain of agricultural remote sensing, U-Net and DeepLab architectures have been extensively validated for the segmentation of small objects, while FCN serves as a natural baseline model for systematic comparison. Furthermore, given that transformer-based models generally necessitate larger training datasets and greater computational resources, the present study focuses on the aforementioned classical and efficient convolutional network architectures to ensure reproducibility, stability, and comparability of experimental results.

#### 3.2.2 Design of the SE channel attention module

To enhance U-Net’s feature representation capabilities in complex field environments, this study introduces a squeeze-excitation (SE) channel attention module. The SE module adaptively recalibrates the importance of feature channels by explicitly modelling inter-channel dependencies (Hu et al., 2017).

**Figure 2:**
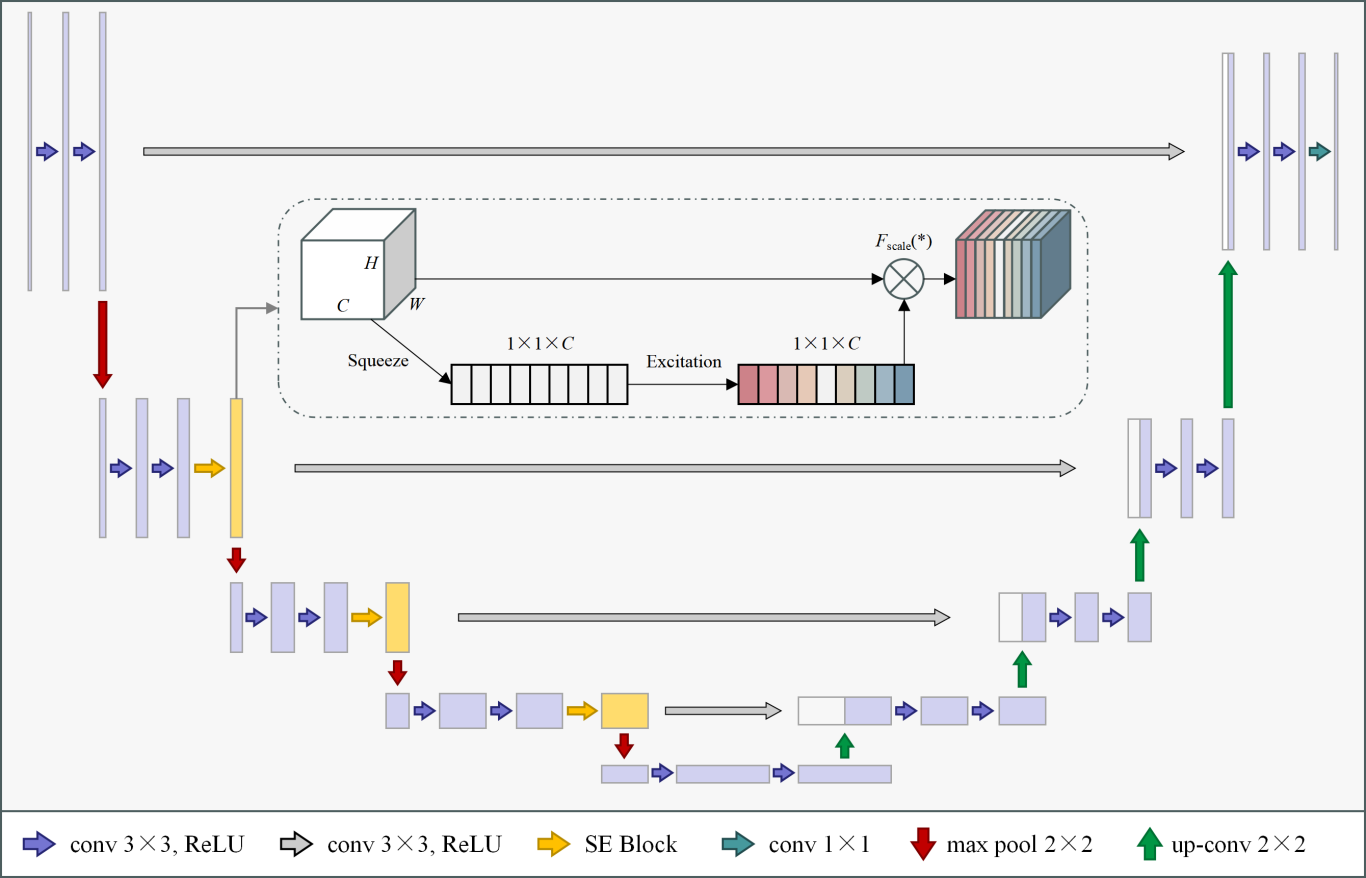
SE-UNet network.

As illustrated in **Figure 2**, the SE module comprises three operations: squeeze, excitation, and scale. First, a global average pooling operation generates channel-level descriptors; subsequently, a two-layer fully connected network learns channel weights; Finally, it recalibrates the original features through per-channel multiplication, amplifying information-rich features while suppressing irrelevant ones.

The SE module is embedded at the end of each stage within the U-Net encoder, enhancing feature recognition of minute seedling regions amidst complex backgrounds. The improved network, termed SE-UNet, undergoes quantitative performance evaluation against baseline U-Net and alternative models in Section 4.

#### 3.2.3 Unified training strategy

In order to present the material clearly and intuitively whilst ensuring comparability between models, the core configurations of the study’s unified training strategy are summarised in the following **Table 1**:

**Table 1:** Unified configuration for model training.

Config item	Parameter setting	Description
Maximum Iterations	40, 000	Validation is performed every 4, 000 iterations.
Optimizer	SGD	Learning rate: 0.0025, Momentum: 0.9, Weight decay: 0.0005
	AdamW	Learning rate: 0.0001, Beta1: 0.9, Beta2: 0.999, Weight decay: 0.01
Learning Rate Schedule	Polynomial decay (PolyLR)	SDG: 1e-4 AdamW: 1e-6
Pre-trained Weights	ResNet-101	The backbone network is initialized with weights pre-trained on ImageNet.
Training Resume	Checkpoint resume mode enabled	Automatically loads the best saved weights to continue training after an interruption.

This standardised configuration serves to eliminate performance variations inherent to the training process itself, thereby ensuring fairness in subsequent model comparisons and the reliability of conclusions drawn.

#### 3.2.4 Data augmentation strategy melting experiment

Image enhancement is crucial for improving the generalisation capability of semantic segmentation models, with different enhancement strategies contributing to segmentation performance to varying degrees. To systematically evaluate the contributions of various enhancement methods, this study designed image enhancement ablation experiments based on the SE-UNet architecture.

In each experiment, only one data enhancement strategy was activated, with all others disabled. This controlled experimental design enables the assessment of each enhancement method’s independent impact on model performance. Three independent experiments were conducted:

1. Scale change only: Random scale transformations (scaling to 0.5–2 times the original image) applied to input images to simulate variations in ground sampling distance and plant size caused by differing flight altitudes.
2. Geometric inversion only: Random horizontal/vertical flipping (flipping probability 0.5) to enhance model robustness against perspective shifts and spatial layout variations.
3. Photometric distortion only: Employed luminance enhancement techniques (random brightness/contrast adjustments: ± 20% brightness, ± 15% contrast) to compensate for illumination variations caused by solar angle shifts and atmospheric conditions during UAV imaging.

By comparing SE-UNet’s segmentation performance under these three independent augmentation strategies, this ablation study aims to reveal the relative effectiveness of scale, geometric, and luminance data augmentation for Kenaf seedling segmentation.

#### 3.2.5 Model testing method

Model testing employs Test Time Augmentation (TTA) technology, generating augmented images through multi-scale transformations and horizontal flipping to enhance prediction accuracy. Given the extended length of the single Image B neighborhood image, a sliding window strategy is adopted for prediction, balancing computational efficiency with accuracy.

Subsequently, SegLocal Visualizer is used to visually compare segmentation results against ground truth labels, enabling intuitive assessment of model performance.

#### 3.2.6 Model evaluation metric

Model performance is comprehensively evaluated using multiple metrics, including: Intersection over Union (IoU), accuracy, F1 score, precision, recall, as well as data loading time and model runtime. The calculation formulas are shown in **Table 2**.

**Table 2:** Formulae for model evaluation metrics.

Abbreviation	Full Name or Calculation Formulas	Serial Number
TP	Positive samples predicted as positive by the model (correctly predicted positive samples)	(1)
TN	Negative samples predicted as negative by the model (correctly predicted negative samples)	(2)
FP	Negative samples predicted as positive by the model (positive samples with incorrect predictions)	(3)
FN	Positive samples predicted as negative by the model (negatively classified samples with incorrect predictions)	(4)
Iou	$\text{IoU}=\frac{\text{TP}}{\text{TP}+\text{FP}+\text{FN}}$	(5)
Accuracy	$\text{Acc}=\frac{\text{TP}+\text{TN}}{\text{TP}+\text{TN}+\text{FP}+\text{FN}}$	(6)
F1-score	$\text{F}1=2*\frac{\text{P}*\text{R}}{\text{P}+\text{R}}$	(7)
Precision	$\text{P}=\frac{\text{TP}}{\text{TP}+\text{FP}}$	(8)
Recall	$\text{R}=\frac{\text{TP}}{\text{TP}+\text{FN}}$	(9)

### 3.3 Calculation of plant canopy coverage during the seedling stage of Kenaf

Based on the optimal segmentation model trained, semantic segmentation was performed on the Image B dataset. The model automatically identified and distinguished the Kenaf canopy coverage from the background areas in the images, generating a binary segmentation result map where plant area pixels are valued as 1 and background pixels as 0.

Subsequently, using NumPy—a Python module for image analysis—the number of pixels within the plant canopy coverage was counted, and its proportion relative to the entire image was calculated. This proportion reflects the coverage of Kenaf in the field image, i.e., the percentage of plant area. The calculation formula is as follows:


Kenaf Canopy Coverage=Number of Pixels Occupied by Plants/Total Number of Pixels×100%

### 3.4 Obtaining Kenaf seedling-stage plant information using remote sensing features

To further investigate the relationship between Kenaf seedling growth information and remote sensing characteristics, this study employed PhenoAI Air software to systematically analyze and extract features from remote sensing data across all experimental plots. This software can remove soil background from images. For each Kenaf variety area, through information annotation, it automatically calculates and outputs 138 remote sensing feature values across three major categories: color features, texture features, and spectral indices (Fu et al., 2023).

Color features primarily reflect the color composition and brightness variations of plant canopies, indirectly indicating crop health status. Texture features describe the spatial distribution patterns of grayscale values in images, aiding in distinguishing plants from soil backgrounds or weeds. Spectral indices quantitatively reflect vegetation coverage, photosynthetic activity, and biomass changes, serving as crucial indicators for vegetation growth monitoring.

**Figure 3:**
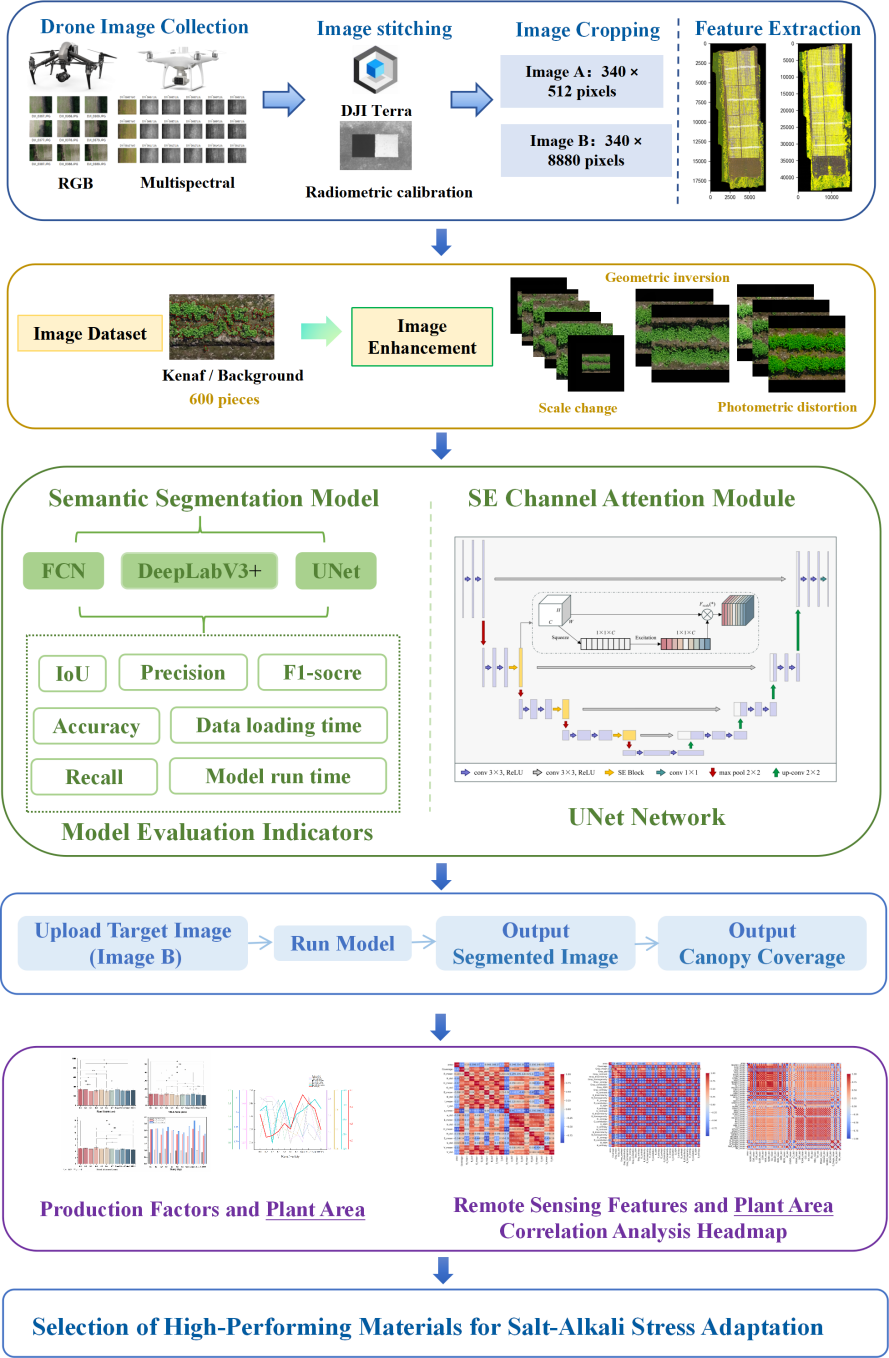
Data collection and processing, model construction and analysis flowchart.

Following feature extraction, multiple feature selection methods, including Pearson correlation analysis, Lasso, Competitive Adaptive Reweighted Sampling (CARS), and Least Angle Regression (LARS), were conducted between various remote sensing feature values and the plant canopy coverage of Kenaf seedlings obtained through optimal model segmentation. By comparing the key features identified through different methods, determine the optimal set of remote sensing feature values. The complete data collection and processing, model construction, and analysis workflow diagram is shown in **Figure 3**.

## 4 Result

### 4.1 Visualization results for model segmentation

To visually demonstrate the performance of different models in segmenting Kenaf seedlings, the segmentation results were visualized. Plant regions are represented in green, while soil background appears in blue. The overlay layer’s opacity was set to 50% to enable simultaneous observation of the original image and segmentation results.

The segmentation visualizations reveal that all three models accurately distinguish Kenaf plants from the soil background, though differences exist in detail handling and boundary recognition. The FCN model achieves overall coherent segmentation with fewer missed detections, yet some soil background misclassification persists within plant regions—particularly in areas with heavy plant shadows. Additionally, when plants densely cluster in small patches, FCN struggles to effectively separate adjacent individuals, leading to minor plant adhesion in small areas.

**Figure 4:**
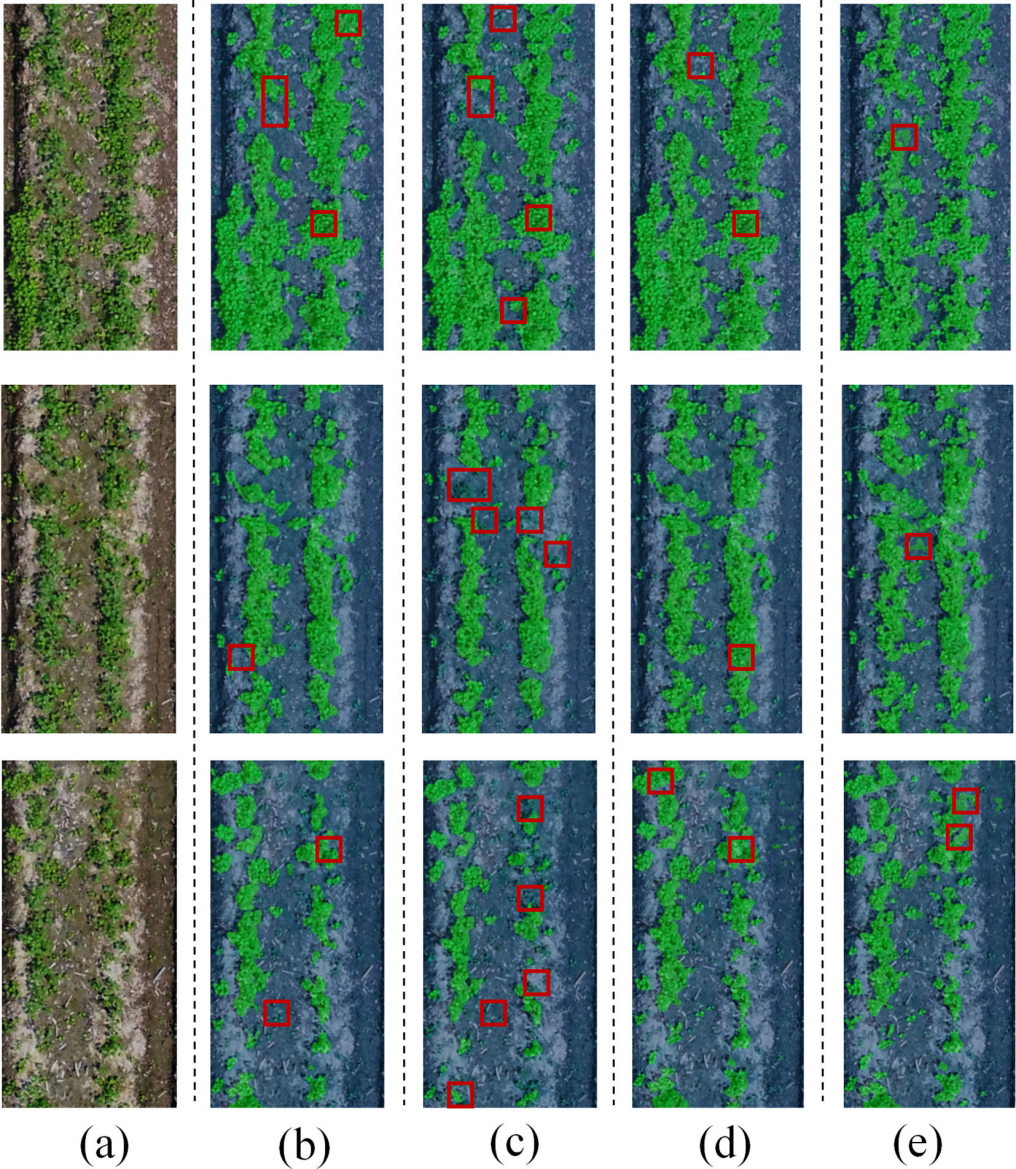
Segmentation results of three different models: **(a)** original image; **(b)** FCN; **(c)** DeepLabv3+; **(d)** U-Net; **(e)** SE-UNet.

The DeepLabv3+ model excels at identifying small-scale individual plants, effectively preserving the morphological features of independent plants. However, it underperforms in recognizing some plants with lighter leaf colors, resulting in a few missed detections (**Figure 4**).

In contrast, the UNet model delivers the best overall segmentation accuracy and regional coherence. Within small areas, UNet preserves plant details more effectively. Although minor missed detections still occur in isolated cases, its overall visual consistency and recognition accuracy surpass those of the other two models.

### 4.2 Accuracy evaluation of three semantic segmentation models

The segmentation results of three models—DeepLabv3+, FCN, and U-Net—were compared (**Table 3**) to evaluate their performance in identifying Kenaf seedlings during the seedling stage. Overall, all three models effectively distinguished Kenaf from soil background areas, though differences existed in accuracy and computational efficiency.

**Table 3:** Segmentation results of three different models.

Model	Type	IoU	Accuracy	F1-score	Precision	Recall	Data loading time	Model run time
DeepLabv3+	Kenaf	78.35	84.34	87.86	91.7	84.34	0.258	1.707
	Background	86.69	95.21	92.87	90.65	95.21		
	Average	82.52	89.77	90.37	91.17	89.77		
FCN	Kenaf	76.71	88.13	86.82	85.54	88.13	0.204	0.566
	Background	84.38	90.66	91.53	92.41	90.66		
	Average	80.54	89.39	89.17	88.98	89.39		
U-Net	Kenaf	81.3	92.33	89.68	87.19	92.33	0.244	0.653
	Background	87.29	91.49	93.21	95.0	91.49		
	Average	84.29	91.91	91.45	91.1	91.91		

Among segmentation accuracy metrics, the U-Net model demonstrated the best performance, achieving an Intersection over Union (IoU) of 84.29%, an average precision of 91.91%, and an F1 score of 91.45%. This indicates that UNet effectively fuses shallow spatial information with deep semantic features through its skip-connection architecture, better preserving Kenaf leaf edge details and texture information, thereby enhancing overall segmentation quality.

The DeepLabv3+ model achieved an average IoU of 82.52%, slightly lower than U-Net, but its precision reached 91.17%, indicating fewer false positives in plant pixel classification. This high precision relates to the ASPP module’s multiscale feature extraction capability, which effectively captures the complex morphology of Kenaf plants. However, DeepLabv3+’s model execution time is approximately 2.5 times longer than the other two models, resulting in relatively low inference efficiency that is less suitable for large-scale rapid processing.

The FCN model achieved an average Intersection over Union (IoU) of 80.54%, performing slightly below the other two models overall. Nevertheless, it maintained a high accuracy rate and stable recall rate. Its simpler model architecture enables the fastest inference speed, making it suitable for applications requiring real-time processing or with limited computational resources.

A comprehensive comparison of the three models reveals that U-Net strikes a favorable balance between segmentation accuracy and computational efficiency. Notably, it achieves the highest recall rate of 92.33% in identifying Kenaf areas, demonstrating its most stable and reliable performance in the task of extracting Kenaf plants.

### 4.3 SE attention and AdamW optimizer synergistically enhance U-net segmentation performance

To enhance and optimise the model architecture, we compared UNet, SE-UNet, and SE-UNet with AdamW (**Table 4**), the SE module consistently achieved improvements across all metrics. mIoU increased by 1.03%, while mF1-score rose by 0.61%. Replacing SGD with AdamW further enhanced SE-UNet’s performance, achieving an overall mIoU improvement of 1.7%, accuracy gain of 1.01%, and mF1-score increase of 0.99%.

**Table 4:** Performance comparison of the U-net model under different configurations.

Model	Optimizer	IoU	Accuracy	F1-score	Precision	Recall
UNet	SDG	84.29	91.91	91.45	91.1	91.91
SE-UNet	SDG	85.32	92.55	92.06	91.68	92.55
SE-UNet	AdamW	85.99	92.92	92.44	92.08	92.92

### 4.4 Data augmentation strategies impact the segmentation performance of SE-UNet

To quantitatively assess the individual contribution of different data augmentation strategies, a series of ablation experiments were conducted using the SE-UNet model optimized with AdamW. In each experiment, only one augmentation strategy was applied while all other augmentation operations were disabled. The segmentation performance under different augmentation configurations is summarized in **Table 5**.

**Table 5:** Performance comparison of data augmentation strategy ablation experiments.

Model	Optimizer	Ablation	IoU	Accuracy	F1-score	Precision	Recall
SE-UNet	AdamW	Scale change	85.08	92.06	91.91	91.77	92.06
SE-UNet	AdamW	Geometric inversion	85.34	92.82	92.07	91.59	92.82
SE-UNet	AdamW	Photometric distortion	85.52	92.93	92.17	91.69	92.93

When scale change was applied, SE-UNet achieved an IoU of 85.08% and an F1-score of 91.91%, indicating that scale-based augmentation can moderately improve the model’s robustness to variations in seedling size and spatial resolution. However, its contribution to overall segmentation accuracy was relatively limited compared with other augmentation strategies.The use of geometric inversion with IoU and F1-score increasing to 85.34% and 92.07%, respectively. Among the tested strategies, photometric distortion resulted in the highest segmentation performance. Under this configuration, SE-UNet achieved an IoU of 85.52%, an accuracy of 92.93%, and an F1-score of 92.17%. These results demonstrate that photometric augmentation is particularly effective in improving model robustness to illumination variability.

### 4.5 Relationship between plant canopy coverage and yield components in different varieties of Kenaf during the seedling stage

In the context of saline-alkali conditions, a notable degree of variability in growth patterns was observed among 11 Kenaf cultivars. The dry bark yield per mu fluctuated between 241.76–538.65 kg/mu, with Xiao 3 and K5 yielding the highest, while K3 yielded the lowest.

**Figure 5:**
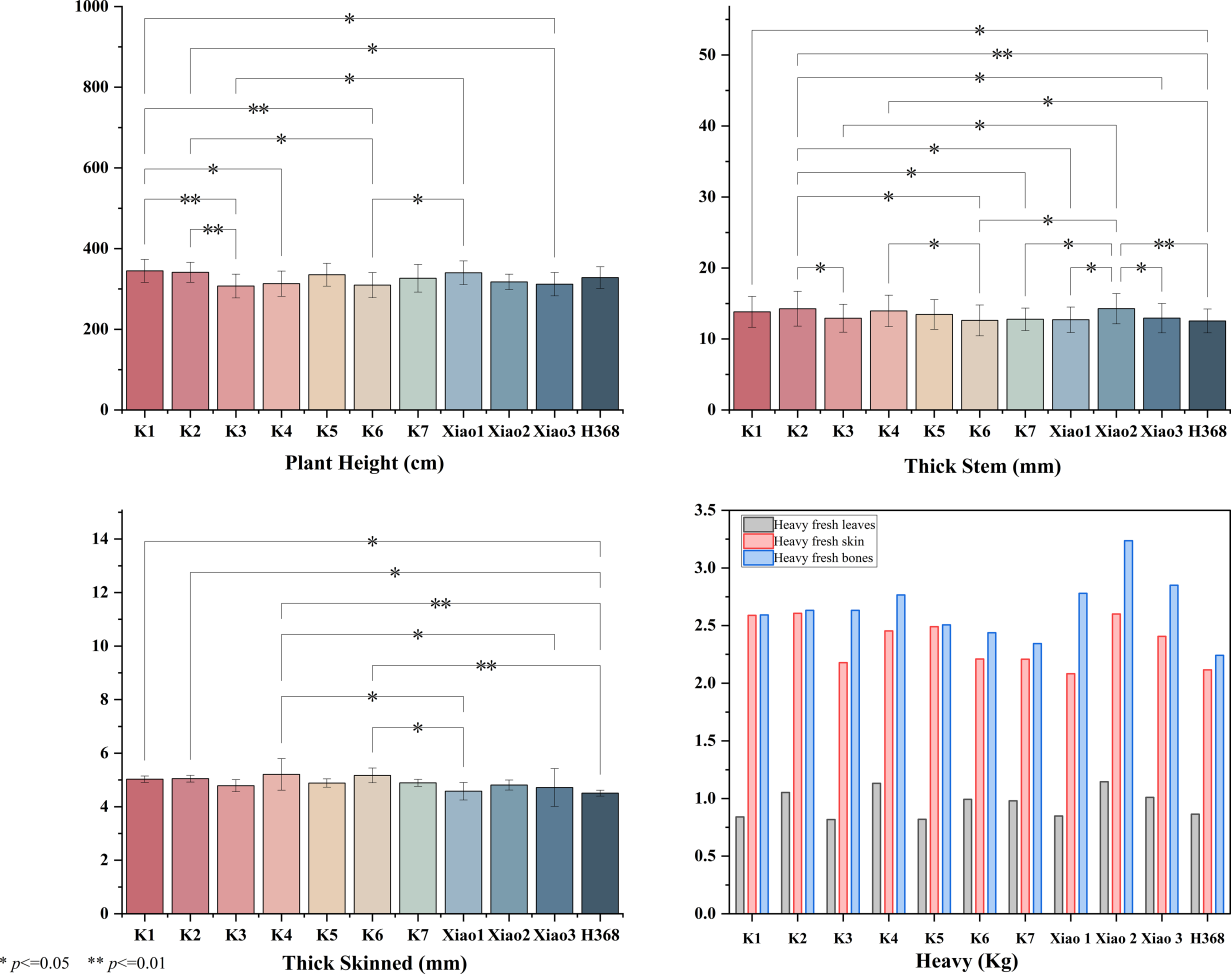
Comparative analysis of yield-related traits among different kenaf varieties.

During the seedling stage, the plant area exhibited marked inter-varietal variation, ranging from 0.2202 to 0.4743. Xiao 1 and Xiao 2 exhibited larger plant areas and vigorous early growth, whereas K2 and K3 displayed smaller plant areas, indicating greater susceptibility to salt stress inhibition during the seedling stage (**Figure 5**).

**Figure 6:**
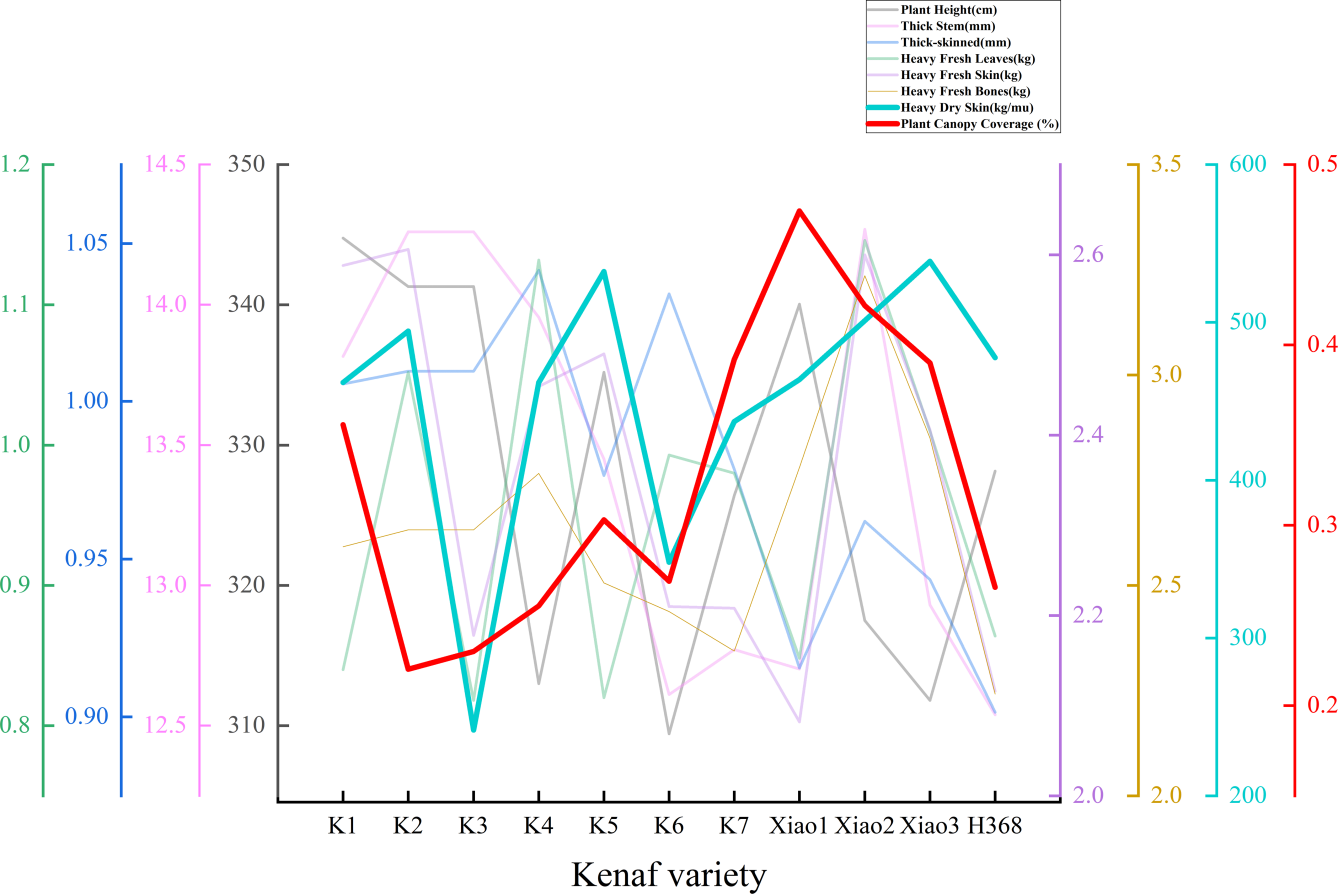
Comparison of plant canopy coverage and yield components of ramie seedlings.

In comparison with conventional morphological indicators, the correlation between seedling-stage plant area and yield demonstrated enhanced stability. The sprout area exhibited a moderately strong positive correlation with dry bark yield (r ≈ 0.52), indicating that early canopy expansion plays a crucial role in subsequent biomass formation (**Figure 6**).

### 4.6 Results of remote sensing feature analysis

To further investigate the relationship between remote sensing characteristics and the growth status of Kenaf seedlings during the seedling stage, a comprehensive feature analysis framework was employed, integrating correlation analysis and multi-method feature selection. The three types of remote sensing characteristics used in this analysis were detailed in our previous study (Fu et al., 2024). The plant canopy coverage derived from optimal semantic segmentation was used as the target variable for subsequent analysis.

**Figure 7:**
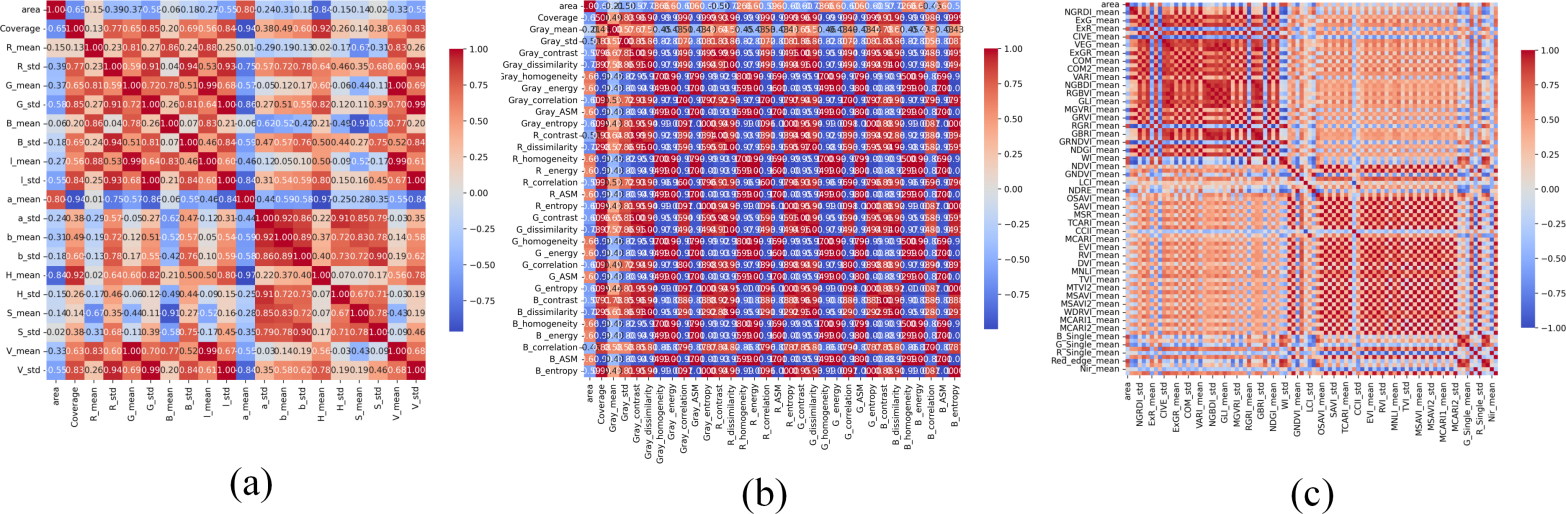
Correlation analysis heatmap: **(a)** Color characteristics; **(b)** Texture characteristics; **(c)** Vegetation characteristics.

Initially, Pearson correlation Analysis results indicate that color characteristics and vegetation index features generally exhibit high correlations with plant area, demonstrating that spectral and color features extracted from imagery can effectively reflect differences in plant canopy coverage and biomass. Among these, 18 feature indicators showed extremely high correlations with plant area (correlation coefficient |r| > 0.8) (**Figure 7**).

These indicators primarily originate from combinations of reflectance values in the visible and near-infrared bands. Vegetation indices such as NDRE, GNDVI, and GRNDVI effectively reflect chlorophyll content and canopy coverage in Kenaf leaves, while color features like ExG, ExGR, and RGRI demonstrate enhanced green characteristics in the visible spectrum, showing significant correlation with plant area (**Table 6**).

**Table 6:** Remote sensing features and their calculation formulas.

Abbreviation	Full Name	Calculation Formulas	Serial Number
B	Blue band	$B=\sum_{i,j}P_{i,j}/N^{2}$	(10)
H	Hue	$H=\frac{1}{N_{p}}\sum_{(i,j)\in mask}P_{H}(i,j)$	(11)
RGRI	Red-Green Ratio Index	RGRI=R/G	(12)
WI_std_	Width Index Standard Deviation	$WI_{std}=\sqrt{\frac{1}{N}\sum_{i=1}^{N}{\left(WI(i)-WI_{mean}\right)}^{2}}$	(13)
NGRDI	Normalized Green-Red Difference Index	NGRDI=(G-R)/(G+R)	(14)
VARI	Visible Atmospherically Resistant Index	VARI=(G-R)/(G+R-B)	(15)
MGVRI	Modified Green-Red Vegetation Index	MGVRI=(G2- R2)/(G2 + R2)	(16)
GRVI	Green-Red Vegetation Index	GRVI=(G-R)/(G+R)	(17)
NDGI	Normalized Difference Greenness Index	NDGI=(G-R)/(G+R)	(18)
GRNDVI	Green-Red Normalized Difference Vegetation Index	GRNDVI=/(NIR/R)*[(NIR-R)/(NIR+R)+1]	(19)
NDRE	Normalized Difference Red Edge Index	NDRE=(NIR-RE)/(NIR+RE)	(20)
WI_mean_	Width Index Mean	$WI_{mean}=\frac{1}{N}\sum_{i=1}^{N}WI(i)$	(21)
a	color channel a average	$a=\frac{1}{N_{p}}\sum_{(i,j)\in mask}p_{a}(i,j)$	(22)
ExR	Excess Red Index	EXR=1.4R-G	(23)
ExGR	Excess Green minus Excess Red Index	ExGR=ExG-1.4R-G	(24)
GNDVI	Green Normalized Difference Vegetation Index	GNDVI=(NIR–G)/(NIR+G)	(25)
G	Green band	$G=\sum_{i,j}P_{i,j}/N^{2}$	(26)
COM	Combination Coefficient	COM=0.25ExG+0.3ExGR+0.33CIVE+0.12VEG	(27)

i denotes the number of rows of pixels, j denotes the number of columns of pixels, P(i, j) denotes the grayscale value at position (i, j) in the image, x and y denote the variances of px(i) and px(j), N denotes the number of rows or columns, and RE, NIR, R, B, G denote the average reflectance of the red edge band, near-infrared band, red band, blue band, and green band respectively.

To more effectively reduce feature redundancy and enhance the robustness of feature selection, this study further integrated three complementary feature selection methods: LASSO, LARS, and CARS. By comprehensively comparing the features selected by different methods, we extracted common key variables, thereby revealing important feature factors capable of stably and consistently predicting plant canopy coverage.

Comparative results demonstrate that features selected by LARS highly overlap with correlation analysis outcomes, including: RGRI, H, WI_mean_, B, a, ExR, WI_std_, and G, indicating these variables exhibit stable strong linear correlations. Similarly, features chosen by CARS—COM, ExR, ExGR, B, a, and NDRE—also share substantial overlap with correlation analysis. Moreover, both LARS and CARS jointly selected the four features ExR, B, S, and a, demonstrating their significant robustness across two distinct feature selection methods. Comprehensive analysis indicates that correlation analysis, LARS, and CARS consistently identified three core features: B, a, and ExR. In contrast, the feature subset selected by LASSO was more streamlined, comprising only ExR, B, and H.

## 5 Discussion

### 5.1 Comparative performance of semantic segmentation models for Kenaf seedlings

This study systematically compares three representative semantic segmentation models—FCN, DeepLabv3+, and U-Net—regarding their efficacy in extracting ramie seedlings from high-resolution aerial imagery. Whilst all models successfully separate seedlings from soil backgrounds, performance variations stem from architectural characteristics and feature representation mechanisms.

As a benchmark end-to-end segmentation framework, FCN demonstrates stable performance and high computational efficiency. However, its limited feature fusion capability constrains its handling of fine boundaries and densely distributed seedlings. DeepLabv3+, leveraging dilated convolutions and multi-scale contextual modelling, excels in small object recognition accuracy. Yet, its high computational complexity prolongs inference time, limiting its application in large-scale or real-time processing.

U-Net achieves the optimal balance between segmentation accuracy and efficiency. Its encoder-decoder architecture with skip connections effectively preserves spatial details while integrating high-level semantic information, making it particularly well-suited for ramie seedling segmentation tasks—a scenario characterised by fragmented plant morphology, significant size variation, and strong background interference. These results confirm U-Net as a reliable benchmark solution for UAV-based crop seedling segmentation tasks.

### 5.2 Effectiveness of SE attention and optimization strategy enhancement

To further enhance segmentation performance, we introduced a squeeze-excited (SE) channel attention module within the UNet architecture. This mechanism enables the network to amplify information-rich spectral and textural channels while suppressing background noise through adaptive calibration of channel-level feature responses. Improvements in intersection-over-union (IoU) and F1 scores demonstrate that channel attention effectively enhances the ability to distinguish small-scale features.

Furthermore, replacing the SGD optimiser with AdamW further elevates model performance. AdamW decouples weight decay from gradient updates, thereby achieving more stable convergence and stronger generalisation capabilities.

### 5.3 Influence of data augmentation strategies on model robustness

Data augmentation ablation experiments demonstrate that different augmentation strategies contribute disparately to segmentation performance. Among the tested strategies, photometric distortion yields the most pronounced improvement, followed by geometric inversion and scale variation. This phenomenon indicates that variability introduced by changing illumination conditions—such as solar angle and shading—constitutes a key source of uncertainty affecting segmentation outcomes in drone-captured images of red hemp seedlings.

### 5.4 Early salt–alkali adaptability and growth structural traits of Kenaf varieties

Under saline-alkali stress, significant variations were observed among different Kenaf varieties in terms of seedling canopy development, stem structure, and yield factors. Varieties exhibiting greater canopy coverage typically demonstrated stronger early growth vigour and higher final dry bark yields, indicating superior early salt tolerance. The contribution of structural traits to yield varied by cultivar: Xiao3 achieved the highest dry bark yield despite possessing medium stem thickness and relatively thin bark; K5 attained high dry bark yield despite lower fresh bark weight, reflecting its outstanding dehydration and biomass conversion efficiency under salt stress. In summary, under the experimental conditions, Xiao3, K5 and Xiao2 demonstrated strong salt tolerance, whereas K3 and K6 exhibited greater sensitivity to salt stress.

### 5.5 Implications of multi-method remote sensing feature selection

Building upon semantic segmentation, this study systematically identified the four key radiometric features most strongly correlated with ramie seedling canopy coverage through correlation analysis and multiple feature selection (LASSO, LARS, CARS): B, a, H, and ExR. The robustness demonstrated by these features across different methodologies supports their potential as reliable phenotypic indicators. Future work may integrate precise segmentation results with the selected features to achieve high-precision, non-destructive monitoring of crop growth in saline-alkali environments.

### 5.6 Research limitations and future prospects

Although this study systematically evaluated three mainstream semantic segmentation models for Kenaf seedling identification, several limitations should be acknowledged. First, the dataset primarily consisted of UAV imagery from a single growth stage, lacking multi-temporal data needed to comprehensively capture dynamic growth patterns. Second, the limited number of training samples and influences such as varying illumination and shadow conditions may have constrained model generalizability. Future research could advance this work in the following directions:

(1) Constructing multi-temporal Kenaf growth monitoring datasets to support time-series growth analysis;
(2) Incorporating emerging network architectures such as Transformer and ConvNeXt to improve recognition performance under complex backgrounds and for small plant objects;
(3) Integrating remote sensing features with meteorological data and field-measured traits to establish accurate yield prediction models for Kenaf.
(4) The utilisation of images captured from multiple angles, such as 30° and 60°, facilitates a more comprehensive documentation of the plant’s additional morphological characteristics, thereby potentially enhancing the accuracy of identification.
(5) Validating Kenaf salt-alkali tolerance by integrating definitive physiological indicators (e.g., ion concentration, photosynthetic efficiency) with image-derived phenotypes to establish a reliable high-throughput phenotyping pipeline.

## 6 Conclusion

This study developed a semantic segmentation framework based on drone imagery for the precise identification and quantification of canopy coverage in Kenaf seedlings within saline-alkali fields. Through systematic comparison of classical convolutional neural network architectures, U-Net was found to achieve the optimal balance between segmentation accuracy, boundary preservation, and computational efficiency, rendering it suitable for extracting early-stage seedling canopy coverage from high-resolution RGB imagery.

Building upon the U-Net baseline model, the introduction of a channel attention mechanism (SE module) and the AdamW optimiser further enhanced the model’s feature discrimination capabilities under complex lighting conditions and background interference. Data augmentation ablation experiments demonstrated that photometric distortion significantly contributed to improved model robustness, underscoring the necessity of employing light-aware training strategies in UAV agricultural applications.

Based on the optimised segmentation results, this study quantitatively extracted seedling canopy coverage and discovered a significant positive correlation with final dry bark yield. This indicates that early canopy development serves as an effective indicator reflecting plant vigour and production potential. Further feature selection employing correlation analysis, LASSO, LARS, and CARS methods consistently identified a small set of key colour-related features: B, a, H, and ExR. The high consistency across different selection methods enhances the reliability of these features for non-destructive phenotyping.

In summary, this study established an accurate and efficient early-stage phenotyping framework for ramie by integrating semantic segmentation, attention mechanism enhancement, data optimisation strategies, and multi-method feature selection. This framework provides a practical and scalable technical solution for drone-based crop growth monitoring in saline-alkali environments, while also offering robust methodological support for precision cultivation and salt-tolerant variety breeding in ramie.

## Data Availability

The original contributions presented in the study are included in the article/supplementary material. Further inquiries can be directed to the corresponding authors.
